# The burden of diabetes and hypertension on healthy life expectancy in Bangladesh

**DOI:** 10.1038/s41598-024-58554-1

**Published:** 2024-04-04

**Authors:** Md. Zakiul Alam, Isna Haque Sheoti

**Affiliations:** 1 Department of Population Sciences, University of Dhaka, Dhaka, 1000 Bangladesh; 2 Department of Population, Family and Reproductive Health, Johns Hopkins Bloomberg School of Public Health, Johns Hopkins University, Maryland, 21205 USA

**Keywords:** Diabetes, Hypertension, Life expectancy, Healthy life expectancy, Diabetes-free life expectancy, Hypertension-free life expectancy, Biomarkers, Diseases, Health care, Medical research, Risk factors, Statistical methods

## Abstract

Diabetes and hypertension are among the leading causes of death in Bangladesh. This study examined hypertension, diabetes, and either or both, free life expectancy, to measure the effect of the diseases on the overall health of individuals in Bangladesh with regional variations. We utilized data from Bangladesh Sample Vital Statistics 2018 for mortality and Bangladesh Demographic and Health Survey 2017–2018 for diabetes and hypertension. The Sullivan method was employed to estimate age-specific hypertension and diabetes-free life expectancy. Altogether, 10.3% of the people aged 18–19 years lived with either diabetes or hypertension. The hypertension-free life expectancy was 40.4 years, and the diabetes-free life expectancy was 53.2 years for those aged 15–19. Overall, individuals would expect to spend 38.7% of their lives with either of the diseases. Females suffered more from hypertension and males from diabetes. Still, females suffered more from the aggregate of both. Rural people had more diabetes and hypertension-free life expectancy than those of urban. Individuals of Mymensingh had the highest life expectancy free of both diseases compared to other divisions of Bangladesh. Diabetes and hypertension affect a considerable proportion of the life of the population in Bangladesh. Policy actions are needed to guide the prevention, diagnosis, and treatment of both diseases, specifically focusing on women and urban populations. Widespread health-enhancing actions need to be taken to diminish the effect of these two diseases in Bangladesh.

## Introduction

Though humanity's greatest ambition of living longer has been achieved in recent decades, that did not come hand in hand with living longer and healthier as decreasing infectious diseases meets up with the increasing number of chronic non-communicable diseases (NCD)^1^. NCD was responsible for 41.4 million deaths in 2017^2^; however, the most considerable effect of NCD is on the long-term impact on the healthy life expectancy of individuals. It is a matter of concern that NCD is increasing rapidly in lower and lower-middle-income countries, which have the potential for more harmful conditions than higher-income countries^3–5^. An estimated 1.28 billion adults aged 30–79 years worldwide have hypertension, and 422 million have diabetes^3,4^.

Studies around the world have found that diabetes and hypertension have substantial impacts on the healthy life expectancy as individuals are living longer with more morbid conditions than in the past^6–14^. Worldwide, from 1990 to 2019, the number of people aged 30–79 with hypertension doubled^15^. Likewise, the total number of people with diabetes increased from 108 million in 1980 to 422 million in 2014^4^. The situation is identical in Bangladesh, and from 2011 to 2018, the prevalence of hypertension increased significantly among both men (20–34%) and women (32–45%)^16^.

Hypertension is when the pressure in the blood vessels is too high (140/90 mmHg or higher)^3^. It is, along with pre-hypertension and other precariously high blood pressure, responsible for 8.5 million deaths from stroke, renal disease, angina, heart attack, irregular heartbeat, ischemic heart disease, and other vascular diseases^17,18^. However, the situation is only the tip of the iceberg, as less than half of adults (42%) with hypertension are diagnosed and treated^3^. Furthermore, Diabetes occurs when the pancreas does not produce enough insulin or the body cannot effectively use the insulin it produces^4^. Diabetes is a fundamental determinant of kidney failure, stroke, blindness, heart attacks, and lower limb amputation. Therefore, it is evident that diabetes and hypertension cause huge damage to individuals and create both short-term and long-term disability for individuals^4,9,14^.

The presence of diabetes and hypertension creates a comorbid situation in individuals^19,20^, where comorbidity is the existence of more than one definite condition or the co-occurrence of several chronic diseases in one person^14,21,22^. Diabetes and hypertension are related as diabetes damages arteries and targets hardening, called atherosclerosis^23^. Hypertension and diabetes together create comorbidity and affect the healthy life expectancy of individuals. Healthy life expectancy is the average years spent in good health that a person would be expected to live, considering the age-specific mortality and morbidity for a given population in a calendar year^5,24,25^.

In Bangladesh, several studies have been conducted regarding healthy life expectancy and the relative contribution of different diseases and disabilities to the healthy life expectancy of the population^26–29^. Those studies have focused on healthy life expectancy and its correlates, gender differences, trends, and active aging index^27,28,30,31^. Many studies have been conducted in Bangladesh to explore the prevalence, socioeconomic variation in the prevalence, association with obesity and overweight, and associated factors^32–40^. For instance, one study has shown that the perceived health of the population improved between 1996 and 2002^26^. Urban and rural areas in Bangladesh have demonstrated different levels of mortality and disability. Bangladesh's rural areas of Bangladesh have experienced higher mortality and morbidity^41^. Another study on disability-free life expectancy in Bangladesh observed gender differences in disability-free life expectancy of old ages in Bangladesh and suggested that women have significantly higher percentages of disabilities than men^29^. Consequently, at all ages, and in both numbers and proportion, women have longer life expectancy but shorter disability-free life expectancy than men.

However, only one study has been conducted to address the relative contribution of hypertension to healthy life expectancy in Bangladesh^42^. No study has been conducted to explore the contribution of diabetes or the aggregate effect of both diseases on the healthy life expectancy of individuals in Bangladesh. It will also be one of the few studies exploring sub-national life expectancy and healthy life expectancy in the context of Bangladesh^28^. Therefore, this study will be one of the original research to measure the effect of comorbidity on healthy life expectancy. This study will estimate to what extent these two diseases (diabetes and hypertension) affect the country's healthy life expectancy with their spatial distribution.

## Methodology

### Data source

Age-sex-specific mortality rates (_n_m_x_) were extracted from published Bangladesh sample vital statistics of 2018^43^. Bangladesh Bureau of Statistics (BBS) initiated the Sample Vital Registration System (SVRS) in 1980 to determine the population change during the intercensal periods. Since the 2013 SVRS, the Integrated Multi-Purpose Sample (IMPS) Design has also been followed^43^. The vital events (e.g., birth, death, marriage, divorce, migration) in the sample area are collected through a dual recording system proposed by Chandrasekaran and Deming^44^. Under a dual record system, vital events are collected (when they occur) by a locally recruited female registrar termed as Local Registrar (System 1). Under a second system (System 2), another group of BBS officials from the same area also collect the data independently. The filled-in questionnaires from the two systems match data in the headquarters by pre-designed matching criteria. The demographic rates and ratios are estimated following Chandrasekaran and Deming's procedure. A household survey is conducted at the beginning of every year to find denominators for the demographic parameters, covering essential household and population characteristics. In 2018, 297,233 households were surveyed from 2012 primary sampling units (PSUs). The total population was 1,259,744, where 630,591 were males and 629,153 females.

On the other hand, the age-sex-specific prevalence of hypertension and diabetes was obtained from the Bangladesh Demographic and Health Survey (BDHS), 2017–2018^16^. BDHS 2017–2018 is the seventh type undertaken in Bangladesh as a part of an international program of measures DHS. The sample for the BDHS is nationally representative, and a detailed methodology will be found elsewhere in the report. The BDHS follows two-stage stratified sampling with a response rate of 98.8%. Blood pressure (BP) and blood glucose (BG) measurement testing were collected from men and women aged 18 years and above in the subsample of 1/4 of the households. All men and women identified as eligible for BP and BG measurements (14,704) were contacted, and the test was explained to them. Testing was taken for those who consented. The measurements were missing for BP for 12.1% of the respondents (16.6% for men and 8.4% for women). The sample size was 12,926 (5583 for men and 7342 for women) for BP. On the other hand, 82.3% (86.3% for women and 77.4% for men) of the respondents had their BG tested. The final sample size for BG stood at 12,100.

### Estimation of hypertension and diabetes

By the LIFE SOURCE® UA-767 Plus BP monitor, BDHS measured the blood pressure^16^. During the interview, three measurements of systolic and diastolic blood pressure (measured in millimeters of mercury [mmHg]) were taken at least 5 min between measurements, using a digital oscillometric blood pressure measuring device with automatic upper-arm inflation and an automatic pressure release. The average of the second and third measurements classified individuals with hypertension. Respondents whose blood pressure fell into two categories based on their mean systolic and diastolic levels were classified according to the highest blood pressure category. If the third measurement was missing, the second measurement was considered the mean. The first measurement was considered the average if the second and third blood pressure measurements were missing. Individuals were classified as hypertensive (coded as 1) if they had an average systolic blood pressure (SBP) level of 140 mmHg or above, they had an average diastolic blood pressure (DBP) level of 90 mmHg or above, or they were currently taking antihypertensive medication, or not (coded as 0).

The diabetes status was calculated based on the fasting plasma glucose (FPG) level. The HemoCue Glucose 201 DM system with plasma conversion was used to test a drop of capillary blood obtained from consenting eligible respondents from the middle or ring finger after fasting overnight. The system automatically converted the survey's fasting whole blood glucose measurements to FPG equivalent values. If individuals had a fasting blood glucose (FBG) equivalent level of 7 mmol/L or above or were currently taking prescribed medication for their high blood glucose or diabetes, they were considered as having raised blood glucose or diabetes (coded as 1) and otherwise as no Diabetes (coded 0).

We also estimated the prevalence of any diabetes or hypertension if the individual suffered from any of two diseases (presence either of the diseases on the individual); the prevalence of both diabetes and hypertension if both were present simultaneously (presence of both diseases on the individual or comorbidity or cooccurring of both diseases).

### Estimation of life expectancy and healthy life expectancy

Healthy life expectancy adds a quality dimension to the quantity of life by splitting life expectancy into years lived with disability or ill health and years lived free of disability or health. It measures whether the increase in life expectancy is years of healthy life or simply extending the lives of the feeble^45^. Using age-specific central death rate (m_x_), we calculated the probabilities of dying (q_x_) using the following formula^46^:

1 $$q_{x} = \frac{{n.m_{x} }}{{1 + \left( {n - a_{x} } \right).m_{x} }}$$

where n is the length of the age group, m_x_ is the central death rate, and a_x_ is the mean number of person-years lived in the interval x to x + n by those dying in the interval. We used a_x_ equals 0.5. Detailed methodology of the life table is available in the book^46^. For instance, we followed the steps given in *Health Expectancy Calculation by the Sullivan Method: A Practical Guide*^47^. Diabetes and Hypertension-free life expectancy has been defined as the number of years spent without those diseases. The following equation calculated hypertension-free life expectancy (HFLE) and diabetes-free life expectancy (DFLE).

2 $$HFLE_{x} = \sum \frac{Lx*Hx}{{lx}}\;{\text{or}}\;DFLE_{x} = \sum \frac{Lx*Dx}{{lx}}$$

where *l*_*x*_ refers to the number of survivors at age x, *L*_*x*_ refers to the person-years lived for the age interval x, *H*_*x*_ refers to the prevalence of hypertension-free for the age interval x, and *D*_*x*_ refers to the prevalence of disability-free for the age interval x. We also calculated the difference in life expectancy by sex. The 95% confidence interval (95% CI) was calculated using Z-test, where p < 0.05 was considered significant. All the analyses were conducted using Microsoft Office 365 and SPSS version 27. All the maps were produced with ArcGIS Pro 2.6.

### Ethics approval and consent to participate

The National Institute of Population Research and Training (NIPORT) of the Ministry of Health and Family Welfare conducted the 2017–18 BDHS. The survey was implemented by a Bangladeshi firm named Mitra and Associates of Bangladesh. At the same time, the ICF International of USA provided technical assistance as part of its international Demographic and Health Surveys Program. If the respondent provided their verbal consent in response to being read out an informed consent statement by the interviewer, only then was an interview conducted. The ethical approval for the survey was taken by the NIPORT from the Bangladesh Medical Research Council (BMRC). This study was carried out in accordance with the Declaration of Helsinki. Moreover, as we used secondary data (published report and de-identified DHS data), approval for this article may not be applicable.

## Findings

### The probability of death, the prevalence of hypertension and diabetes

**Figure 1:**
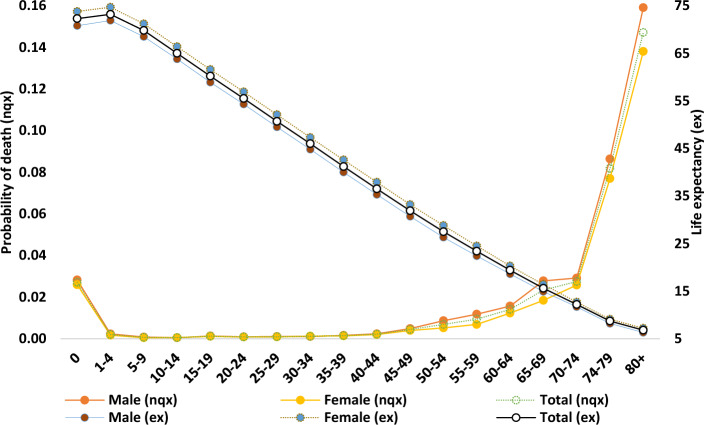
Age-sex-specific probability of dying (_n_q_x_) and life expectancy (e_x_) in Bangladesh, 2018.

The probability of age-specific probability of dying and life expectancy are presented in Fig. 1 (regional variation of age-specific mortality and life expectancy was provided in Supplementary Tables 1–2, 9). Life expectancy also varied with age; at ages 15–19, life expectancy ranged from 67 to 71 years, while at ages 1–4 years, it ranged from 69 to 72 years. The life expectancy of 1–4 years was higher than that of 0–1 years. Besides this, all life expectancy decreased with age as mortality increased exponentially.

The immediate mortality risk at any age was observed to follow an exponential curve with age. The probability of dying was high at an early age and increased with aging. Life expectancy also varied with age; at ages 15–19, life expectancy ranged from 67 to 71 years, while at ages 1–4 years, it ranged from 69 to 72 years. The life expectancy of 1–4 years was higher than that of 0–1 years. Besides this, all life expectancy decreased with age as mortality increased exponentially.

Table 1 represents the prevalence of hypertension and diabetes among the adult population in Bangladesh in 2017–18. The prevalence of hypertension among aged 18–19 was 6.8%. Males were more prevalent than females in this age group (10.6% vs 5.0%). The prevalence of hypertension increased with age and was highest at ages 80 or above. The prevalence of diabetes was 3.8% among ages 18–19 in Bangladesh. Again, males had a higher prevalence of diabetes than females in this age group (5.7% vs 2.9%). Diabetes is most prevalent among the middle age group (50–54 years). Divisional age-specific prevalence of hypertension and diabetes was provided in Supplementary Tables 3–4 and 10.

**Table 1:** Prevalence of hypertension and diabetes among the adult population (aged 18 years and above) in Bangladesh, 2017–18.

Age group	Hypertension			Diabetes			Either hypertension or diabetes			Both hypertension and diabetes		
	Male	Female	Total	Male	Female	Total	Male	Female	Total	Male	Female	Total
18–19	10.6	5.0	6.8	5.7	2.9	3.8	15.7	7.8	10.3	0.0	0.0	0.0
20–24	12.8	7.6	9.5	5.1	3.7	4.2	16.3	11.4	13.1	1.8	0.1	0.6
25–29	11.5	14.0	13.0	3.6	5.6	4.9	14.0	18.3	16.7	0.7	1.1	1.0
30–34	16.8	21.3	19.5	6.9	8.0	7.6	22.0	25.7	24.3	1.1	3.2	2.4
35–39	22.1	31.4	27.0	8.8	11.6	10.3	28.6	38.4	33.8	3.1	4.6	3.9
40–44	22.0	37.3	30.3	13.7	12.5	13.0	31.3	44.1	38.3	5.2	5.9	5.6
45–49	28.3	43.4	36.9	11.7	15.2	13.6	34.2	49.7	43.0	6.0	9.2	7.8
50–54	34.9	47.5	40.8	17.9	17.3	17.6	45.8	54.2	49.7	7.0	11.8	9.3
55–59	37.6	51.0	45.0	14.0	16.8	15.6	45.2	55.2	50.7	7.3	11.8	9.8
60–64	43.6	54.0	48.6	18.3	15.2	17.0	53.0	60.2	56.4	9.3	11.0	10.1
65–69	48.3	55.0	51.2	16.4	13.3	15.0	55.6	59.9	57.5	11.3	9.2	10.4
70–74	51.0	52.7	51.7	19.3	15.5	17.8	57.9	58.4	58.1	12.0	9.4	10.9
74–79	44.8	62.7	52.6	12.9	14.8	13.8	51.8	67.2	57.9	7.1	11.5	9.0
80 +	57.3	68.8	63.5	15.3	11.4	13.3	64.9	73.6	69.4	9.0	8.1	8.5

Prevalence data was only available from age group 18 in BDHS^48^. Prevalence of ages 0–17 had not been reported because of lower prevalence reported by past studies^49,50^.

The percentage of the population with either hypertension or diabetes was 10.3% among age group 18–19. Again, males outnumbered females in the prevalence (15.7% vs 7.8%). The prevalence of both diseases was the highest in the age group 70–74, with overall prevalence of 10.9%. Males were more prevalent than females in that age group (12% vs 9.4%).

### Hypertension-free life expectancy

Table 2 presents hypertension-free life expectancy in Bangladesh with a 95% confidence interval. Males had a more hypertension-free life expectancy (42 years) than females (38.8 years) for those aged 15–19 years. It is coherent with the previous Table 1, as we have seen that the prevalence of hypertension is higher among females. The percentage of years lived with hypertension was 32.8 for those aged 15–19 years old.

**Table 2:** Hypertension-free life expectancy with 95 confidence intervals (CI) in Bangladesh, 2018.

Age	Hypertension free life expectancy (95% CI				% of years lived with hypertension, total (male, female)
	Male	Female	Difference	Total	
0	54.7	51.9	2.8	53.3	26.3 (22.8, 29.7)
1–4	55.2	52.2	3.0	53.7	24.6 (23.2, 30.1)
5–9	51.7	48.6	3.1	50.2	25.9 (24.5, 31.8)
10–14	46.9	43.7	3.2	45.3	27.8 (26.5, 34.2)
15–19	42.0 (41.3, 42.7)	38.8 (38.0, 39.6)	3.2 (1.7, 4.7)***	40.4 (39.9, 41.0)	32.8 (28.7, 37.0)
20–24	37.8 (37.1, 38.5)	34.2 (33.4, 35.0)	3.6 (2.1, 5.0)***	36.0 (35.5, 36.5)	35.1 (30.4, 39.8)
25–29	33.6 (32.9, 34.3)	29.7 (29.0, 30.5)	3.9 (2.4, 5.3) ***	31.6 (31.1, 32.1)	37.6 (32.1, 42.9)
30–34	29.3 (28.7, 30.0)	25.6 (24.8, 26.3)	3.8 (2.3, 5.2)***	27.4 (26.9, 27.9)	40.4 (34.5, 46.0)
35–39	25.3 (24.6, 26.0)	21.8 (21.0, 22.5)	3.5 (2.1, 5.0)***	23.5 (23.0, 24.)	42.9 (36.7, 48.9)
40–44	21.6, (21.0, 22.3)	18.5 (17.7, 19.2)	3.1 (1.7, 4.6)***	20.0 (19.5, 20.5)	45.1 (38.8, 51.3)
45–49	17.9 (17.3, 18.6)	15.5 (14.8, 16.3)	2.4 (1.1, 3.8)***	16.8 (16.3, 17.2)	47.5 (41.6, 53.3)
50–54	14.8 (14.1, 15.4)	13.0 (12.2, 13.7)	1.8 (0.4, 3.2)**	13.9 (13.5, 14.4)	49.3 (43.9, 55.0)
55–59	12.1 (11.5, 12.7)	10.6 (9.9, 11.3)	1.4 (0.1, 2.7)*	11.4 (10.9, 11.9)	51.3 (46.1, 56.6)
60–64	9.6 (9.0, 10.2)	8.5 (7.8, 9.2)	1.1 (− 0.2, 2.3)	9.1 (8.7, 9.6)	52.9 (48.4, 58.1)
65–69	7.4 (6.9, 8.0)	6.7 (6.0, 7.4)	0.8 (− 0.5, 2.0)	7.1 (6.7, 7.6)	54.3 (50.1, 59.3)
70–74	5.8 (5.2, 6.3)	5.0 (4.3, 5.6)	0.8 (− 0.4, 2.0)	5.4 (5.0, 5.8)	55.6 (50.8, 60.9)
74–79	4.0 (3.5, 4.6)	3.1 (2.5, 3.7)	0.9 (− 0.2, 2.0)	3.6 (3.2, 4.0)	58.5 (51.1, 65.9)
80 +	2.7 (2.2, 3.2)	2.3 (1.7, 2.8)	0.4 (− 0.6, 1.4)	2.5 (2.1, 2.8)	63.5 (57.4, 68.6)

z ≤ 0.001 = ***, ≤ 0.01 = **, ≤ 0.05 = *; we could not estimate CI for ages 0–14. Blood pressure and diabetes for age group 15–19 presented in the data is for age group 18–19 extracted from BDHS. We assume it is for the whole age group.

### Diabetes-free life expectancy

Table 3 presents the Diabetes free life expectancy with 95 percent confidence intervals in Bangladesh. The prevalence of diabetes was lower than hypertension and more likely to occur at a later age. The years spent without diabetes were 53.2 years (54.5 years for females, and 52.1 years for males aged 15–19), with 11.6% of years expected to live with diabetes.

**Table 3:** Diabetes-free life expectancy with 95 confidence intervals (CI) in Bangladesh, 2018.

Age	Diabetes free life expectancy (95% CI)				% of years lived with diabetes, total (male, female)
	Male	Female	Difference	Total	
0	64.4	67.0	− 2.7	65.6	9.3 (9.1, 9.1)
1–4	65.2	67.8	− 2.6	66.4	9.3 (9.3, 9.2)
5–9	61.8	64.2	− 2.5	62.9	9.9 (9.8, 9.8)
10–14	57.0	59.4	− 2.4	58.1	10.6 (10.6, 10.6)
15–19	52.1 (51.6, 52.7)	54.5 (54.0, 55.1)	− 2.4 (− 3.5, − 1.3) ***	53.2 (52.9, 53.6)	11.6 (11.5, 11.5)
20–24	47.7 (47.2, 48.3)	50.0 (49.4, 50.6)	− 2.2 (− 3.3, − 1.1) ***	48.7 (48.4, 49.1)	12.2 (12.1, 12.2)
25–29	43.2 (42.7, 43.7)	45.4 (44.8, 45.9)	− 2.1 (− 3.3, − 1.1) ***	44.1 (43.8, 44.5)	12.9 (12.7, 13.0)
30–34	38.6 (38.1, 39.1)	40.8 (40.2, 41.4)	− 2.2 (− 3.3, − 1.1) ***	39.6 (39.2, 40.0)	13.9 (13.8, 13.7)
35–39	34.1 (33.6, 34.6)	36.5 (35.9, 37.0)	− 2.3 (− 3.4, − 1.2) ***	35.2 (34.8, 35.6)	14.6 (14.7, 14.4)
40–44	29.8 (29.3, 30.3)	32.3 (31.7, 32.8)	− 2.4 (− 3.5, − 1.4) ***	30.9 (30.6, 31.3)	15.2 (15.5, 14.9)
45–49	25.8 (25.3, 26.3)	28.2 (27.6, 28.7)	− 2.4 (− 3.4, − 1.3) ***	26.9 (26.5, 27.3)	15.6 (15.8, 15.1)
50–54	22 (21.5, 22.5)	24.5 (23.9, 25.0)	− 2.5 (− 3.5, − 1.4) ***	23.1 (22.8, 23.5)	15.8 (16.4, 15.0)
55–59	18.8 (18.3, 19.2)	20.9 (20.4, 21.4)	− 2.2 (− 3.1, − 1.2) ***	19.8 (19.4, 20.1)	15.6 (16.2, 14.6)
60–64	15.5 (15.0, 15.9)	17.4 (16.9, 17.9)	− 1.9 (− 2.9, − 1.0) ***	16.4 (16.0, 16.7)	15.6 (16.8, 14.2)
65–69	12.5 (12.1, 12.9)	14.1 (13.6, 14.6)	− 1.7 (− 2.6, − 0.7) ***	13.3 (12.9, 13.6)	15.0 (16.3, 13.8)
70–74	9.8 (9.4, 10.3)	11.0 (10.5, 11.4)	− 1.1 (− 2.0, − 0.2) *	10.4 (10.1, 10.7)	14.9 (15.8, 13.7)
74–79	7.0 (6.6, 7.4)	7.9 (7.6, 8.4)	− 0.9 (− 1.7, − 0.1) *	7.5 (7.2, 7.8)	14.0 (14.3, 12.8)
80+	5.3 (4.9, 5.7)	6.4 (6.0, 6.8)	− 1.1 (− 1.8, − 0.3)	5.9 (5.6, 6.2)	13.3 (15.5, 10.9)

z ≤ 0.001 = ***, ≤ 0.01 = **, ≤ 0.05 = *; we could not estimate CI for ages 0–14. Blood pressure and diabetes for age group 15–19 presented in the data is for age group 18–19 extracted from BDHS. We assume it is for the whole age group.

### Either hypertension or diabetes-free life expectancy

Table 4 presents either hypertension or diabetes-free (absence of any of diseases) life expectancy with 95 confidence intervals (CI). Males were anticipated to spend 38.1 years of their life with either of the disease-free at age 15–19. On the other hand, the females would spend 35.6 years either hypertension or diabetes-free for the same age. Overall, individuals would expect to spend 38.7% of their lives with either of the diseases.

**Table 4:** Either hypertension or diabetes-free life expectancy with 95 confidence intervals (CI) in 2018.

Age	Either hypertension or diabetes free life expectancy (95% CI)				% of years lived with either, total (male, female)
	Male	Female	Difference	Total	
0	50.9	48.8	2.1	49.9	31.0 (28.1, 33.9)
1–4	51.4	49.1	2.3	50.3	31.3 (28.6, 34.3)
5–9	47.8	45.4	2.4	46.7	33.2 (30.2, 36.3)
10–14	43.0	40.5	2.5	41.8	35.7 (32.6, 39.0)
15–19	38.1 (37.3, 38.9)	35.6 (34.8, 36.4)	2.5 (0.9, 4.1) **	36.9 (36.3, 37.4)	38.7 (35.3, 42.2)
20–24	34.1 (33.4, 34.9)	31.2 (30.4, 32.0)	3.0 (1.4, 4.5)***	32.6 (32.1, 33.2)	41.2 (37.2, 45.2)
25–29	30.1 (29.3, 30.8)	26.9 (26.1, 27.7)	3.2 (1.7, 4.8)***	28.4 (27.9, 28.9)	44.0 (39.2, 48.5)
30–34	25.9 (25.2, 26.7)	22.9 (22.1, 23.7)	3.1 (1.5, 4.6)***	24.4 (23.8, 24.9)	47.0 (42.1, 51.6)
35–39	22.2 (21.5, 22.9)	19.3 (18.5, 20.1)	2.9 (1.4, 4.4)***	20.7 (20.2, 21.2)	49.7 (44.6, 54.7)
40–44	18.7 (18.1, 19.4)	16.3 (15.5, 17.1)	2.4 (1.0, 3.9)**	17.5 (17.0, 18.1)	52.0 (46.9, 56.9)
45–49	15.5 (14.8, 16.2)	13.7 (12.9, 14.4)	1.8 (0.4, 3.3)**	14.6 (14.1, 15.1)	54.2 (49.5, 58.8)
50–54	12.6 (11.9, 13.2)	11.4 (10.7, 12.2)	1.1 (− 0.2, 2.5)*	12.1 (11.6, 12.6)	56.1 (52.2, 60.4)
55–59	10.3 (9.7, 11.0)	9.4 (8.7, 10.1)	1.0 (− 0.4, 2.3)	9.9 (9.5, 10.4)	57.6 (53.8, 61.7)
60–64	8.1 (7.6, 8.7)	7.4 (6.7, 8.1)	0.7 (− 0.6, 2.0)	7.9 (7.4, 8.3)	59.4 (56.2, 63.4)
65–69	6.4 (5.8, 6.9)	5.9 (5.2, 6.5)	0.5 (− 0.8, 1.8)	6.2 (5.7, 6.6)	60.3 (57.3, 64.3)
70–74	4.9 (4.4, 5.5)	4.3 (3.7, 5.0)	0.6 (− 0.6, 1.8)	4.7 (4.3, 5.1)	61.5 (57.3, 66.0)
74–79	3.4 (2.9, 4.0)	2.7 (2.1, 3.3)	0.7 (− 0.4, 1.9)	3.1 (2.7, 3.5)	64.0 (58.4, 70.6)
80 +	2.2 (1.7, 2.7)	1.9 (1.4, 2.4)	0.3 (− 0.7, 1.3)	2.1 (1.7, 2.4)	69.4 (65.0, 73.5)

z ≤ 0.001 = ***, ≤ 0.01 = **, ≤ 0.05 = *; we could not estimate CI for for age 0–14. Blood pressure and diabetes for age group 15–19 presented in the data is for age group 18–19 extracted from BDHS. We assume it is for the whole age group.

### Both hypertension and diabetes-free life expectancy

Table 5 presents both hypertension and diabetes-free life expectancy with a 95 percent confidence interval. On average, the population aged 15–19 spends 6% of their total life span with both diseases. The females spend more years with both hypertension and diabetes-free (57.5 years) than males (55.8 years).

**Table 5:** Both hypertension and diabetes-free life expectancy with 95 confidence intervals (CI).

Age	Both hypertension and diabetes free life expectancy (95% CI)				% of years lived with both, total (male, female)
	Male	Female	Difference	Total	
0	67.9	69.9	− 2.0	68.8	4.8 (4.1, 5.2)
1–4	68.8	70.7	− 1.9	69.7	4.8 (4.3, 5.3)
5–9	65.5	67.2	− 1.8	66.3	5.1 (4.4, 5.6)
10–14	60.7	62.4	− 1.7	61.5	5.4 (4.8, 6.1)
15–19	55.8 (55.5, 56.2)	57.5 (57.0, 58.0)	− 1.7 (− 2.5, − 0.8)***	56.6 (56.3, 56.9)	6.0 (5.2, 6.6)
20–24	51.2 (50.8, 51.6)	52.8 (52.4, 53.3)	− 1.6 (− 2.5, − 0.8)***	51.9 (51.6, 52.2)	6.4 (5.7, 7.1)
25–29	46.5 (46.1, 46.9)	48.0 (47.6, 48.5)	− 1.5 (− 2.4, − 0.7)***	47.2 (46.9, 47.5)	6.9 (6.1, 7.8)
30–34	41.8 (41.4, 42.2)	43.3 (42.8, 43.8)	− 1.5 (− 2.4, − 0.7)***	42.4 (42.1, 42.7)	7.7 (6.8, 8.5)
35–39	37.0 (36.6, 37.4)	38.7 (38.2, 39.2)	− 1.7 (− 2.6, − 0.8)***	37.8 (37.5, 38.1)	8.3 (7.4, 9.1)
40–44	32.5 (32.1, 32.8)	34.2 (33.7, 34.7)	− 1.7 (− 2.6, − 0.9)***	33.2 (32.9, 33.5)	8.9 (8.1, 9.8)
45–49	28.1 (27.7, 28.4)	29.8 (29.3, 30.3)	− 1.7 (− 2.6, − 0.9)***	28.9 (28.6, 29.2)	9.5 (8.6, 10.3)
50–54	24.0 (23.6, 24.4)	25.8 (25.3, 26.3)	− 1.8 (2.6, − 1.0)***	24.8 (24.5, 25.1)	9.7 (8.7, 10.4)
55–59	20.3 (19.9, 20.7)	22.0 (21.6, 22.5)	− 1.7 (− 2.5, − 0.9)***	21.1 (20.8, 21.4)	9.9 (9.4, 10.2)
60–64	16.8 (16.4, 17.1)	18.3 (17.8, 18.7)	− 1.5 (− 2.3, − 0.7)***	17.5 (17.2, 17.8)	9.9 (9.9, 9.9)
65–69	13.4 (13.0, 13.7)	14.8 (14.4, 15.3)	− 1.5 (− 2.2, − 0.7)***	14.1 (13.8, 14.4)	9.7 (10.1, 9.5)
70–74	10.6 (10.3, 10.9)	11.5 (11.1, 11.9)	− 0.9 (− 1.6, − 0.2)*	11.1 (10.8, 11.3)	9.3 (9.2, 9.3)
74–79	7.5 (7.2, 7.8)	8.2 (7.8, 8.6)	− 0.7 (− 1.4, 0.0)*	7.9 (7.7, 8.1)	9.2 (8.2, 9.5)
80+	5.7 (5.4, 6.0)	6.7 (6.3, 7.0)	− 0.9 (− 1.6, − 0.3)**	6.2 (6.0, 6.4)	8.5 (9.2, 7.5)

z ≤ 0.001 = ***, ≤ 0.01 = **, ≤ 0.05 = *; we could not estimate CI for age 0–14; Blood pressure and diabetes for age group 15–19 presented in the data is for age group 18–19 extracted from BDHS. We assume it is for the whole age group.

### The urban–rural difference in Diabetes and Hypertension-free life expectancy

**Figure 2:**
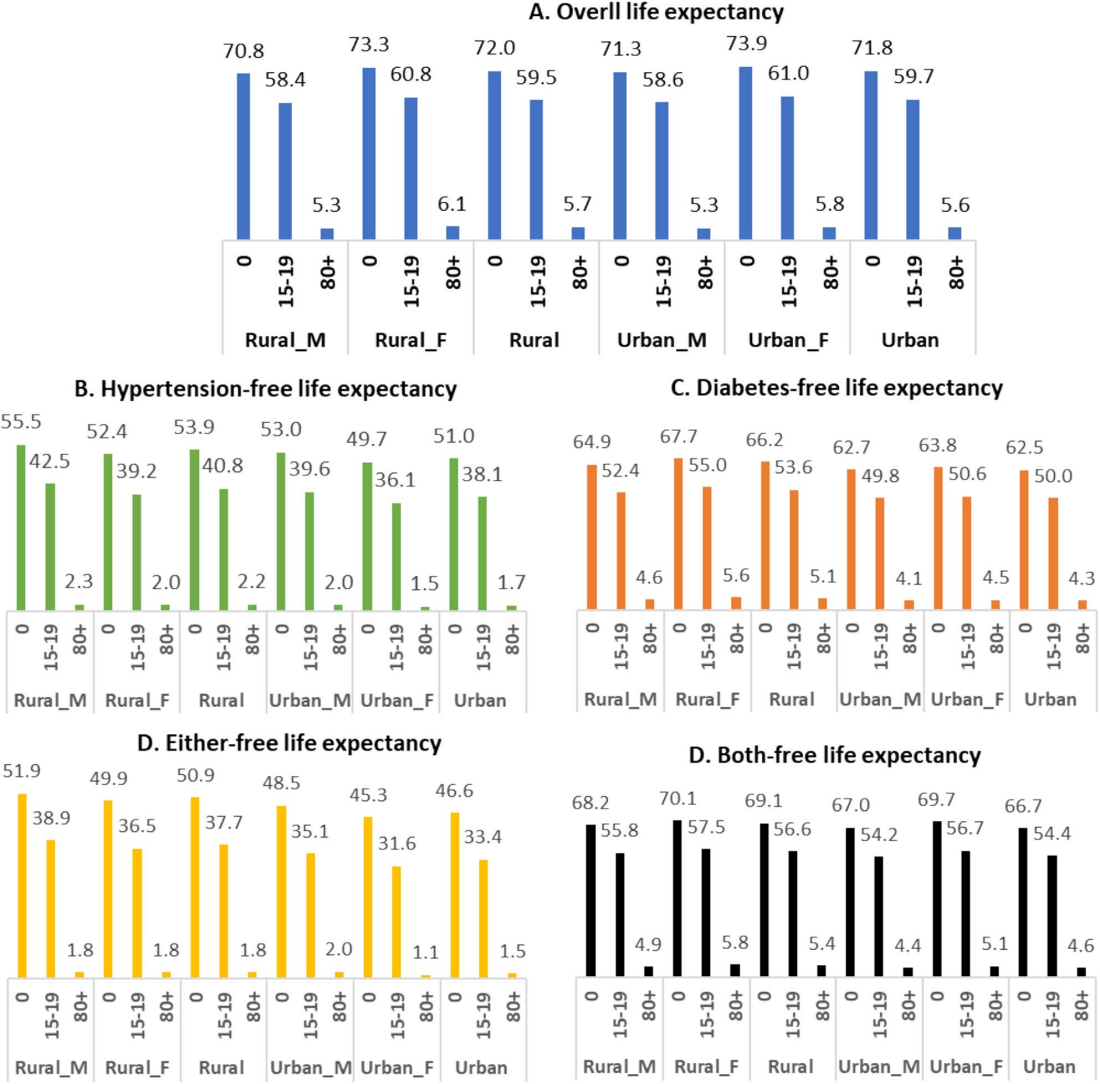
Diabetes and Hypertension free life expectancy by residence in Bangladesh.

Figure 2 presents diabetes and hypertension-free life expectancy among urban and rural populations in Bangladesh. The rural population spends more years free of disability (Hypertension, Diabetes, either of them, or both). While the rural population (aged 15–19) spent 56.6 years without both diseases, the number was 37.7 years for either of the diseases. Moreover, urban and rural males had more hypertension-free life expectancy, whereas urban and rural females had more diabetes-free life expectancy. The detailed findings on diabetes and hypertension-free life expectancy were added to Supplementary Table 11.

### The divisional difference in diabetes and hypertension-free life expectancy

**Figure 3:**
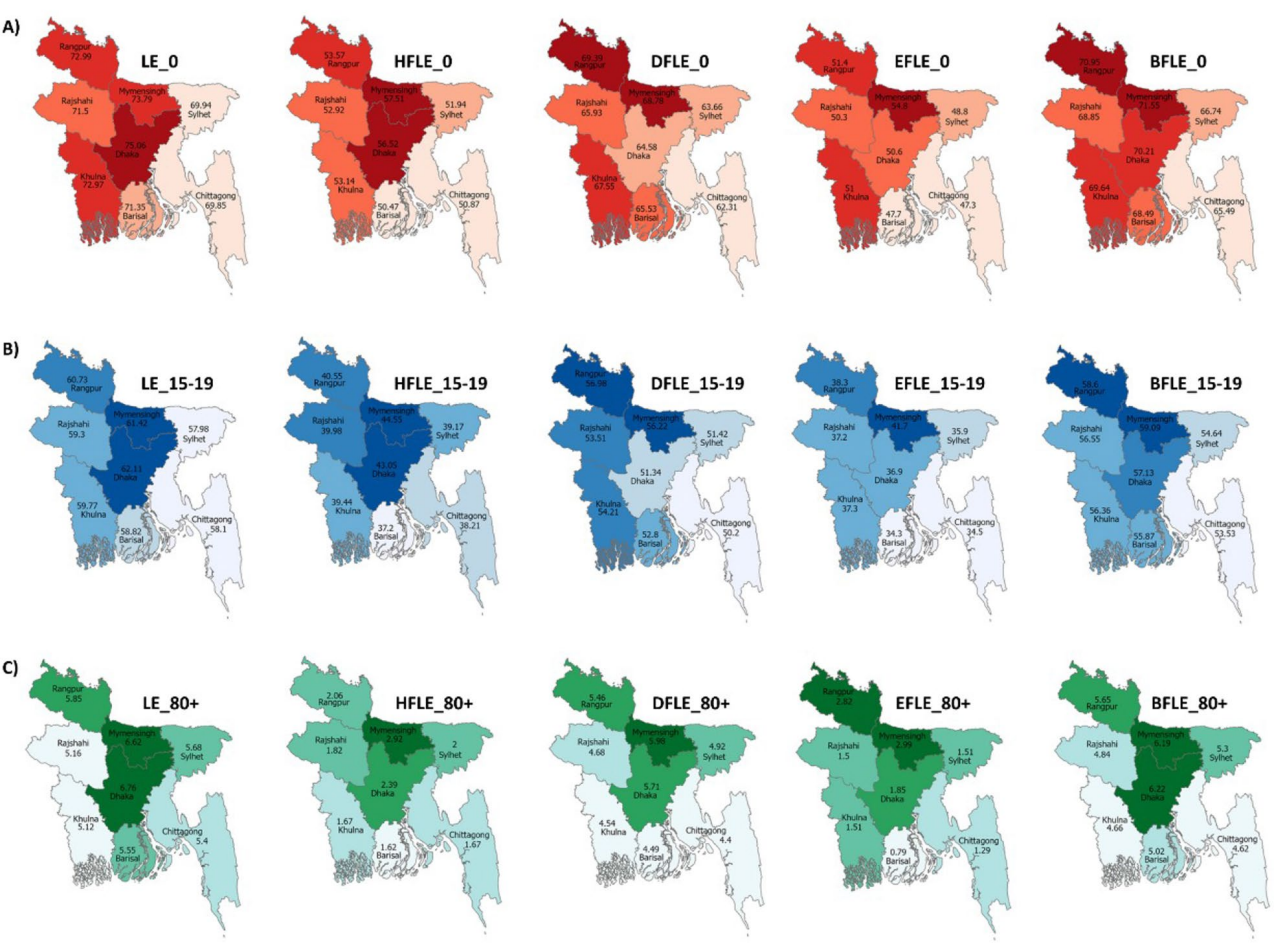
Divisional difference in diabetes and hypertension-free life expectancy in Bangladesh [**A**: Life expectancy at birth (LE_0_, HFLE_0,_, DFLE_0_, EFLE_0_, BFLE_0_); **B**: Life expectancy at age 15–19 years (LE_15–19_, HFLE_15–19,_, DFLE_15–19_, EFLE_15–19_, BFLE_15–19_); **C**: Life expectancy at aged 80 and above (LE_80+_, HFLE_80+,_, DFLE_80+_, EFLE_80+_, BFLE_80+_)]. LE: Life expectancy, HFLE: Hypertension-free life expectancy, DFLE: Diabetes-free life expectancy, EFLE: Either Hypertension or Diabetes free life expectancy, BFLE: Both Hypertension and Diabetes free life expectancy].

Figure 3 shows the divisional difference in diabetes and hypertension-free life expectancy in Bangladesh using a series of maps. The measurements of hypertension-free life expectancy, diabetes-free life expectancy, either hypertension or diabetes-free life expectancy, and both hypertension and diabetes-free life expectancy are presented in supplementary tables (STable 5–8). The brighter color in the maps indicates more hypertension and diabetes-free life expectancy. Hypertension-free life expectancy was the highest in Mymensingh (57.5) and the lowest in Barishal (50.5). Rangpur had the highest diabetes-free life expectancy (69.4), and Chattogram had the lowest (62.3). While Mymensingh (54.8) had the highest either hypertension or diabetes-free life expectancy, Chattogram (47.3) had the lowest. Mymensingh (71.6) had the highest life expectancy free of both diseases, and Chattogram had the lowest (65.5).

## Discussions

The study aimed to explore the contribution of hypertension and diabetes to healthy life expectancy reduction in Bangladesh. The prevalence of hypertension and diabetes is relatively high in Bangladesh. On average, a person aged 15–19 is expected to live 32.8% of their life with hypertension in Bangladesh. Whereas the life expectancy of those ages 15–19 was 58.9 years in 2018 in Bangladesh, they would spend 40.4 years without hypertension. Almost 18.5 years were expected to live with hypertension. These findings correlate with other study findings^51,52^. The main reason for this high prevalence of hypertension in Bangladesh is the increasing prevalence of obesity, tobacco use, high intake of processed foods, salt intake, and less physical activity^51,52^. Overall, the life expectancy of ages 1–4 years was higher than that of 0–1-year due to higher infant mortality in Bangladesh.

Our study found that males spent more years being hypertension-free than females. Several studies found the reasons for females' higher prevalence of hypertension, including higher prevalence of obesity, use of oral contraception, preeclampsia, and menopause, male and female differences in functional status, nutritional status, inadequate health care service availability for women, patriarchy, and culture^53–56^. Women are outliving men in Bangladesh, which also works as a reason why women spend more years living with hypertension^42^.

On the contrary, the prevalence of diabetes is less acute than hypertension. The number of years spent without diabetes was 53.2 years. Males and females had different patterns in diabetes when compared to hypertension. Females spent more years without diabetes. A higher prevalence of type 2 diabetes in men than in women was associated with differences in Visceral Fat Mass as males stored more fat in their bellies^57,58^. In different studies, physical activity, smoking, and indifference to testing the disease were shown as reasons^59,60^.

Furthermore, either hypertension or diabetes-free life expectancy presents the burden of both diseases on the population. Again, females spent fewer years (35.6 years) without either disease than 38.1 years for males. Around 39% of life is expected to be lived with either disease in Bangladesh, whereas 6% of females spent more years with both diseases. This pattern was also evident in other literature, indicating the life expectancy advantages of women in Bangladesh as *Failures of success*^28^*.* Increasing life expectancy would come with chronic disease, economic insolvency, poor mental health, and misery. The lifelong discrimination and patriarchy were becoming evident with disease patterns^28^.

Both diabetes and hypertension are more prevalent with increasing age. The percentage of years lived with both hypertension and diabetes increased with age. This finding was also consistent with another study, as the prevalence of infectious diseases increases with age^23,51,52,61^. Our study found that hypertension-free life expectancy for age group 0 is 53.3 years, and diabetes-free life expectancy is 65.6 years. On the other hand, the hypertension-free life expectancy for the age group 10–14 was 45.3 years, and 58.1 years for diabetes-free life expectancy. Again, both hypertension and free life expectancy decreased substantially with age. The prevalence of diabetes^49^ and hypertension^50^ among children (0–14 years of age) is low in Bangladesh, and it is also true for many other countries^62–68^.

Moreover, our study found that the prevalence of diabetes and hypertension was higher in urban areas than in rural areas in almost all age groups. The rural population had more years free of disability (hypertension or diabetes). Studies around the world presented that urban individual showed a higher prevalence of hypertension and diabetes than their rural counterparts^69–72^. However, studies in America demonstrated that the prevalence of hypertension and diabetes was higher among both white and black rural populations^54,73^. In Bangladesh, previous studies indicated that the urban population had a higher prevalence of hypertension and diabetes than the rural population^74–76^.

This study found that the population from Mymensingh and Rangpur divisions had the highest hypertension and diabetes-free life expectancy in Bangladesh. The poverty level of the areas can explain this; Rangpur and Mymensingh had the highest poverty rate in Bangladesh^77^. Previous studies of Bangladesh and other lower-income countries presented that higher wealth status had a positive relationship with the prevalence of hypertension, diabetes, and other non-communicable diseases and their risk factors^32,78–80^.

## Strengths and limitations

This study has some important strengths. This study discovered the effects of both diseases on life expectancy in Bangladesh. It was crucial to measure it as it would increase the burden of non-communicable diseases for the population with increasing life expectancy. The Sullivan method for calculating healthy life expectancy has some important strengths. Applying data from cross-sectional studies was straightforward and less influenced by survey design and analytic strategies than methods relying on longitudinal data.

The limitations of the methods include the method's assumptions constraining the portrayal of the expected life cycle or functional status histories of persons exposed to current mortality and morbidity conditions. It does not permit recovery once individuals have experienced a health problem. It will yield an inaccurate portrayal of the timing and volume of a cohort's health experiences when individuals experience the onset of health problems and recovery. Although the Sullivan method could not detect a sudden change in health problems, it provides relatively stable estimates as the multistate life table method if there are smooth and relatively regular changes in health problem prevalence rates over a long time^81,82^.

We used a limited number of social determinants for life expectancy due to the unavailability of data in Bangladesh. Finally, we have estimated the prevalence of diabetes and hypertension in the age group 18–19 from BDHS. However, we calculated hypertension-free life expectancy, diabetes-free life expectancy, either hypertension or diabetes-free life expectancy, and both free life expectancy from age 0. The life expectancy of the earlier ages is influenced by the life expectancy of later ages (later mortality or morbidity) in the life table, a synthetic cohort^46^. Therefore, it may not be wise to construct different life tables for different age groups and analyze only a subsection of the population.

Moreover, one previous study in Bangladesh has presented that the prevalence of hypertension among school-going children was 1.8% (male was 1.68% and female was 1.99%)^50^, and another study presented that the prevalence of diabetes mellitus was 1.8%^49^. Thus, the prevalence of diabetes and hypertension among the younger population is very low in Bangladesh. As a result, assuming no diabetes and hypertension among those aged 0–14 (and considering the rate of 18–19 for 15–19) may not substantially affect the estimation as the prevalence of diabetes and hypertension among the younger population is very low in Bangladesh. Despite the above issues, this study would be worth mentioning to present recent regional variations in life expectancy.

## Conclusions and implications

The world is experiencing increasing life expectancy due to improved socioeconomic conditions and medical and public health advancements, and Bangladesh is no exception. With increasing life expectancy, non-communicable diseases and their impact on later life have become more significant. This study provides insights into diabetes and hypertension-free life expectancy in Bangladesh. Diabetes and hypertension are becoming more prevalent day by day in the country. These two diseases are affecting the healthy life expectancy of individuals, and they also contribute to other diseases. In these circumstances, policies and programs need to reduce the prevalence of diabetes and hypertension. Efforts must be made from the early stages of life through physical activity, food patterns, salt taking, mental health, and other factors. Mass screening programs need to be enforced to detect taking steps early and appropriately. The focus should be on cost-effective and readily available treatment for both diseases. Women suffer more from both diseases. Emphasis should be given to women as they live more than males. This study has brought forward this quality-of-life dimension with the quantity dimension of life expectancy. This study will ignite policymakers' and people's consciousness about the burden of diabetes and hypertension.

### Supplementary Information

Supplementary Information.

## Data Availability

The 2017–18 BDHS data set is anonymized and freely available at https://dhsprogram.com/data/, and the instructions were followed for using the data. The published Bangladesh sample vital statistics of 2018 is available at the Bangladesh Bureau of Statistics (BBS) website: http://www.bbs.gov.bd/site/page/ef4d6756-2685-485a-b707-aa2d96bd4c6c/.
