# Supplementary material for: The burden of diabetes and hypertension on healthy life expectancy in Bangladesh
Source: Sci Rep. 2024 Apr 4;14:7936. doi: 10.1038/s41598-024-58554-1 (PMC10995204; doi:10.1038/s41598-024-58554-1)
Supplement: Supplementary file 1 — Supplementary Information. [file 41598_2024_58554_MOESM1_ESM.docx]

**Supplementary Table**

**STable 1**: Age-specific mortality rate (%) by Division in Bangladesh, 2018

| **Age** | **Barishal** | **Chattogram** | **Dhaka** | **Khulna** | **Rajshahi** | **Rangpur** | **Sylhet** | **Mymensingh** |
| --- | --- | --- | --- | --- | --- | --- | --- | --- |
| **0** | 2.44 | 3.56 | 1.86 | 1.77 | 3.06 | 2.85 | 3.37 | 2.81 |
| **1-4** | 0.47 | 0.60 | 0.38 | 0.44 | 0.57 | 0.54 | 0.56 | 0.57 |
| **5-9** | 0.09 | 0.09 | 0.06 | 0.04 | 0.04 | 0.04 | 0.07 | 0.04 |
| **10-14** | 0.05 | 0.05 | 0.06 | 0.03 | 0.05 | 0.06 | 0.05 | 0.02 |
| **15-19** | 0.17 | 0.11 | 0.06 | 0.11 | 0.14 | 0.10 | 0.20 | 0.06 |
| **20-24** | 0.10 | 0.10 | 0.08 | 0.06 | 0.06 | 0.12 | 0.06 | 0.11 |
| **25-29** | 0.12 | 0.11 | 0.08 | 0.09 | 0.06 | 0.08 | 0.15 | 0.04 |
| **30-34** | 0.09 | 0.18 | 0.08 | 0.10 | 0.09 | 0.12 | 0.13 | 0.13 |
| **35-39** | 0.12 | 0.16 | 0.13 | 0.14 | 0.21 | 0.15 | 0.07 | 0.25 |
| **40-44** | 0.27 | 0.21 | 0.24 | 0.25 | 0.09 | 0.15 | 0.39 | 0.17 |
| **45-49** | 0.42 | 0.66 | 0.42 | 0.36 | 0.41 | 0.35 | 0.54 | 0.42 |
| **50-54** | 0.67 | 0.76 | 0.45 | 0.56 | 0.86 | 0.77 | 0.86 | 0.53 |
| **55-59** | 1.07 | 1.18 | 0.55 | 1.00 | 0.96 | 0.76 | 1.22 | 0.89 |
| **60-64** | 1.56 | 1.63 | 1.05 | 1.23 | 1.49 | 1.45 | 1.83 | 0.90 |
| **65-69** | 2.32 | 2.77 | 1.81 | 2.50 | 2.72 | 1.75 | 2.75 | 1.92 |
| **70-74** | 3.87 | 3.29 | 2.67 | 2.20 | 2.42 | 1.89 | 3.07 | 2.42 |
| **75-79** | 9.00 | 9.01 | 6.31 | 9.20 | 8.49 | 7.53 | 8.55 | 6.84 |
| **80+** | 11.26 | 11.78 | 8.40 | 12.81 | 12.66 | 10.39 | 10.87 | 8.66 |

**STable 2**: Divisional life expectancy in Bangladesh, 2018

| **Age** | **Barishal** | **Chattogram** | **Dhaka** | **Khulna** | **Rajshahi** | **Rangpur** | **Sylhet** | **Mymensingh** |
| --- | --- | --- | --- | --- | --- | --- | --- | --- |
| **0** | 71.3 | 69.8 | 75.1 | 73.0 | 71.5 | 73.0 | 69.9 | 73.8 |
| **1-4** | 72.0 | 71.2 | 75.4 | 73.2 | 72.6 | 74.0 | 71.2 | 74.8 |
| **5-9** | 68.4 | 67.7 | 71.7 | 69.5 | 69.0 | 70.4 | 67.6 | 71.2 |
| **10-14** | 63.7 | 62.9 | 66.9 | 64.7 | 64.1 | 65.5 | 62.8 | 66.4 |
| **15-19** | 58.8 | 58.1 | 62.1 | 59.8 | 59.3 | 60.7 | 58.0 | 61.4 |
| **20-24** | 54.3 | 53.4 | 57.3 | 55.1 | 54.7 | 56.0 | 53.5 | 56.6 |
| **25-29** | 49.6 | 48.7 | 52.5 | 50.2 | 49.9 | 51.3 | 48.7 | 51.9 |
| **30-34** | 44.8 | 43.9 | 47.7 | 45.5 | 45.0 | 46.5 | 44.0 | 47.0 |
| **35-39** | 40.0 | 39.3 | 42.9 | 40.7 | 40.2 | 41.8 | 39.3 | 42.3 |
| **40-44** | 35.3 | 34.6 | 38.2 | 35.9 | 35.6 | 37.1 | 34.4 | 37.8 |
| **45-49** | 30.7 | 29.9 | 33.6 | 31.4 | 30.7 | 32.4 | 30.1 | 33.1 |
| **50-54** | 26.3 | 25.8 | 29.2 | 26.9 | 26.3 | 27.9 | 25.8 | 28.7 |
| **55-59** | 22.1 | 21.7 | 24.9 | 22.6 | 22.4 | 23.9 | 21.8 | 24.4 |
| **60-64** | 18.2 | 17.9 | 20.5 | 18.6 | 18.4 | 19.7 | 18.1 | 20.4 |
| **65-69** | 14.5 | 14.2 | 16.4 | 14.6 | 14.6 | 16.0 | 14.6 | 16.3 |
| **70-74** | 10.9 | 11.0 | 12.8 | 11.3 | 11.3 | 12.2 | 11.3 | 12.6 |
| **75-79** | 7.6 | 7.5 | 9.2 | 7.3 | 7.5 | 8.2 | 7.8 | 9.0 |
| **80+** | 5.6 | 5.4 | 6.8 | 5.1 | 5.2 | 5.8 | 5.7 | 6.6 |

**STable 3**: Age-specific hypertension rate (%) by Division in Bangladesh, 2018

| **Age** | **Barishal** | **Chattogram** | **Dhaka** | **Khulna** | **Rajshahi** | **Rangpur** | **Sylhet** | **Mymensingh** |
| --- | --- | --- | --- | --- | --- | --- | --- | --- |
| **18-19** | 8.7 | 7.4 | 3.9 | 8.3 | 8.5 | 9.4 | 7.8 | 3.0 |
| **20-24** | 11.4 | 10.5 | 7.3 | 8.0 | 16.1 | 7.9 | 7.0 | 8.5 |
| **25-29** | 11.6 | 19.5 | 10.6 | 10.5 | 8.5 | 16.7 | 17.7 | 9.3 |
| **30-34** | 20.2 | 24.8 | 16.7 | 22.7 | 18.7 | 24.2 | 13.2 | 13.5 |
| **35-39** | 29.1 | 33.8 | 25.3 | 24.5 | 24.2 | 29.9 | 20.4 | 25.2 |
| **40-44** | 33.3 | 33.5 | 27.8 | 35.1 | 23.1 | 34.2 | 29.4 | 26.7 |
| **45-49** | 42.2 | 40.9 | 36.6 | 36.3 | 36.8 | 40.5 | 37.7 | 21.7 |
| **50-54** | 44.7 | 42.7 | 39.3 | 41.2 | 45.8 | 40.7 | 36.6 | 31.1 |
| **55-59** | 46.2 | 49.6 | 38.8 | 48.9 | 44.5 | 50.5 | 46.0 | 34.3 |
| **60-64** | 54.5 | 51.1 | 45.7 | 47.6 | 47.0 | 52.0 | 54.3 | 39.4 |
| **65-69** | 70.0 | 44.4 | 52.9 | 40.7 | 47.3 | 59.0 | 55.2 | 53.3 |
| **70-74** | 56.3 | 45.0 | 49.4 | 61.0 | 71.1 | 39.6 | 60.0 | 41.9 |
| **75-79** | 72.7 | 48.1 | 35.5 | 77.3 | 45.0 | 50.0 | 60.0 | 52.6 |
| **80+** | 70.8 | 69.1 | 64.6 | 67.4 | 64.7 | 44.8 | 64.7 | 55.9 |

[Note: Prevalence data was only available from age group 18 in BDHS. ^48^ Prevalence of ages 0-17 had not been reported because of lower prevalence reported by past studies.^49,50^]

**STable 4**: Age-specific diabetes rate (%) by Division in Bangladesh, 2018

| **Age** | **Barishal** | **Chattogram** | **Dhaka** | **Khulna** | **Rajshahi** | **Rangpur** | **Sylhet** | **Mymensingh** |
| --- | --- | --- | --- | --- | --- | --- | --- | --- |
| **18-19** | 2.4 | 2.3 | 7.4 | 1.1 | 4.5 |  | 4.8 | 4.9 |
| **20-24** | 6.7 | 3.7 | 8.0 | 0.6 | 1.4 | 2.9 | 3.3 | 3.5 |
| **25-29** | 3.8 | 4.5 | 6.0 | 3.7 | 5.8 | 0.5 | 8.6 | 5.9 |
| **30-34** | 9.1 | 7.2 | 10.8 | 9.1 | 4.3 | 4.5 | 9.3 | 5.7 |
| **35-39** | 12.0 | 12.2 | 17.6 | 6.0 | 4.8 | 6.0 | 8.0 | 8.5 |
| **40-44** | 5.2 | 11.2 | 23.3 | 11.9 | 12.3 | 6.7 | 13.8 | 9.9 |
| **45-49** | 10.3 | 21.4 | 20.3 | 9.2 | 9.0 | 8.3 | 15.4 | 7.6 |
| **50-54** | 16.7 | 23.6 | 19.7 | 18.9 | 17.3 | 8.1 | 15.8 | 14.3 |
| **55-59** | 23.1 | 22.0 | 18.3 | 13.2 | 11.4 | 8.6 | 16.7 | 13.3 |
| **60-64** | 17.1 | 24.0 | 27.4 | 9.8 | 13.1 | 9.3 | 12.2 | 12.7 |
| **65-69** | 13.8 | 21.4 | 19.4 | 10.5 | 21.2 | 10.0 | 14.8 | 6.8 |
| **70-74** | 6.5 | 11.8 | 33.3 | 21.8 | 18.6 | 11.3 | 10.0 | 10.5 |
| **75-79** | NA | 16.7 | 24.1 | 9.5 | 10.5 | 7.1 | 20.0 | 6.3 |
| **80+** | 19.0 | 18.4 | 15.6 | 11.4 | 9.4 | 6.7 | 13.3 | 9.7 |

[Note: Prevalence data was only available from age group 18 in BDHS. ^48^ Prevalence of ages 0-17 had not been reported because of lower prevalence reported by past studies.^49,50^]

**STable 5**: Divisional hypertension-free life expectancy in Bangladesh, 2018

| **Age** | **Barishal** | **Chattogram** | **Dhaka** | **Khulna** | **Rajshahi** | **Rangpur** | **Sylhet** | **Mymensingh** |
| --- | --- | --- | --- | --- | --- | --- | --- | --- |
| **0** | 50.5 (70.7) | 50.9 (72.8) | 56.5 (75.3) | 53.1 (72.8) | 52.9 (74.0) | 53.6 (73.4) | 51.9 (74.3) | 57.5 (77.9) |
| **1-4** | 50.7 (70.3) | 51.6 (72.4) | 56.6 (75.0) | 53.1 (72.5) | 53.5 (73.7) | 54.0 (73.0) | 52.6 (73.9) | 58.1 (77.6) |
| **5-9** | 46.9 (68.6) | 47.9 (70.8) | 52.8 (73.6) | 49.3 (70.9) | 49.8 (72.1) | 50.3 (71.5) | 48.9 (72.3) | 54.4 (76.4) |
| **10-14** | 42.1 (66.1) | 43.1 (68.5) | 47.9 (71.6) | 44.4 (68.6) | 44.9 (70.0) | 45.4 (69.3) | 44.1 (70.1) | 49.5 (74.6) |
| **15-19** | 37.2 (63.2) | 38.2 (65.8) | 43.0 (69.3) | 39.4 (66.0) | 40.0 (67.4) | 40.6 (66.8) | 39.2 (67.6) | 44.5 (72.5) |
| **20-24** | 32.9 (60.6) | 33.8 (63.3) | 38.4 (67.0) | 35.1 (63.6) | 35.7 (65.2) | 36.2 (64.6) | 34.9 (65.3) | 39.8 (70.4) |
| **25-29** | 28.7 (57.8) | 29.5 (60.6) | 33.9 (64.5) | 30.6 (60.8) | 31.6 (63.3) | 32.2 (62.7) | 30.4 (62.4) | 35.5 (68.3) |
| **30-34** | 24.4 (54.4) | 25.6 (58.3) | 29.5 (61.9) | 26.2 (57.6) | 27.1 (60.2) | 27.7 (59.6) | 26.5 (60.1) | 31.0 (65.9) |
| **35-39** | 20.5 (51.2) | 22.0 (56.1) | 25.5 (59.4) | 22.5 (55.2) | 23.1 (57.6) | 23.8 (57.0) | 22.3 (56.7) | 26.9 (63.5) |
| **40-44** | 17.1 (48.4) | 18.9 (54.6) | 21.9 (57.4) | 18.8 (52.4) | 19.6 (55.0) | 20.2 (54.4) | 18.4 (53.4) | 23.4 (62.0) |
| **45-49** | 14.0 (45.4) | 15.8 (52.6) | 18.5 (55.2) | 15.8 (50.4) | 15.8 (51.4) | 16.5 (50.9) | 15.2 (50.5) | 20.0 (60.3) |
| **50-54** | 11.3 (43.1) | 13.3 (51.4) | 15.7 (53.8) | 12.9 (47.9) | 12.9 (49.2) | 13.6 (48.7) | 12.4 (48.2) | 16.4 (57.1) |
| **55-59** | 8.9 (40.3) | 10.9 (50.0) | 13.0 (52.4) | 10.3 (45.4) | 10.7 (48.0) | 11.4 (47.6) | 9.8 (44.7) | 13.4 (54.7) |
| **60-64** | 6.6 (36.4) | 8.9 (49.9) | 10.3 (50.2) | 8.2 (43.9) | 8.4 (45.9) | 9.0 (45.5) | 7.6 (42.0) | 10.6 (52.0) |
| **65-69** | 4.8 (33.2) | 7.2 (50.3) | 8.0 (48.9) | 6.0 (40.9) | 6.3 (43.4) | 6.9 (43.1) | 5.9 (40.7) | 8.0 (49.2) |
| **70-74** | 3.8 (34.7) | 5.2 (47.7) | 6.3 (49.6) | 3.6 (32.1) | 4.4 (39.0) | 4.8 (39.0) | 4.4 (38.7) | 6.4 (50.3) |
| **75-79** | 2.1 (28.1) | 3.2 (42.3) | 4.5 (49.0) | 2.0 (27.1) | 3.5 (46.2) | 3.7 (45.4) | 2.9 (37.8) | 4.1 (45.7) |
| **80+** | 1.6 (29.2) | 1.7 (30.9) | 2.4 (35.4) | 1.7 (32.6) | 1.8 (35.3) | 2.1 (35.3) | 2.0 (35.3) | 2.9 (44.1) |

**Note**: Percentage of hypertension-free life expectancy in the parenthesis

**STable 6**: Divisional diabetes-free life expectancy in Bangladesh, 2018

| **Age** | **Barishal** | **Chattogram** | **Dhaka** | **Khulna** | **Rajshahi** | **Rangpur** | **Sylhet** | **Mymensingh** |
| --- | --- | --- | --- | --- | --- | --- | --- | --- |
| **0** | 65.5 (91.8) | 62.3 (89.2) | 64.6 (86.0) | 67.5 (92.6) | 65.9 (92.2) | 69.4 (95.1) | 63.7 (91.0) | 68.8 (93.2) |
| **1-4** | 66.1 (91.7) | 63.4 (89.1) | 64.8 (85.9) | 67.7 (92.5) | 66.9 (92.1) | 70.3 (95.0) | 64.7 (90.9) | 69.7 (93.1) |
| **5-9** | 62.4 (91.2) | 59.8 (88.4) | 61.0 (85.1) | 64.0 (92.0) | 63.3 (91.6) | 66.7 (94.7) | 61.1 (90.4) | 66.0 (92.7) |
| **10-14** | 57.7 (90.6) | 55.1 (87.5) | 56.2 (83.9) | 59.1 (91.4) | 58.4 (91.0) | 61.8 (94.3) | 56.3 (89.6) | 61.2 (92.2) |
| **15-19** | 52.8 (89.8) | 50.2 (86.4) | 51.3 (82.7) | 54.2 (90.7) | 53.5 (90.2) | 57.0 (93.8) | 51.4 (88.7) | 56.2 (91.5) |
| **20-24** | 48.3 (89.0) | 45.6 (85.3) | 46.9 (81.8) | 49.6 (90.0) | 49.1 (89.8) | 52.3 (93.3) | 47.2 (88.1) | 51.6 (91.2) |
| **25-29** | 43.9 (88.6) | 41.0 (84.2) | 42.4 (80.8) | 44.7 (89.0) | 44.3 (88.9) | 47.7 (92.9) | 42.4 (87.2) | 47.1 (90.7) |
| **30-34** | 39.4 (87.8) | 36.4 (82.9) | 37.9 (79.4) | 40.1 (88.2) | 39.7 (88.3) | 42.9 (92.2) | 38.2 (86.7) | 42.5 (90.4) |
| **35-39** | 35.0 (87.4) | 32.1 (81.7) | 33.6 (78.3) | 35.7 (87.9) | 35.1 (87.3) | 38.4 (91.8) | 33.9 (86.2) | 38.0 (89.9) |
| **40-44** | 30.8 (87.3) | 27.9 (80.8) | 29.7 (77.7) | 31.3 (87.0) | 30.7 (86.2) | 34.0 (91.5) | 29.4 (85.4) | 33.9 (89.7) |
| **45-49** | 26.4 (86.0) | 23.8 (79.4) | 26.2 (77.9) | 27.2 (86.8) | 26.4 (86.0) | 29.5 (91.2) | 25.6 (85.2) | 29.6 (89.6) |
| **50-54** | 22.4 (85.3) | 20.6 (79.6) | 22.7 (77.6) | 23.2 (86.1) | 22.4 (85.1) | 25.4 (91.2) | 22.0 (85.3) | 25.6 (89.1) |
| **55-59** | 19.0 (85.8) | 17.5 (80.3) | 19.1 (77.0) | 19.7 (87.2) | 19.1 (85.6) | 21.7 (91.0) | 18.7 (85.6) | 22.0 (89.8) |
| **60-64** | 16.1 (88.3) | 14.5 (81.0) | 15.5 (75.9) | 16.3 (87.3) | 15.6 (84.8) | 17.9 (90.9) | 15.6 (86.3) | 18.5 (90.6) |
| **65-69** | 13.1 (90.2) | 11.8 (82.8) | 12.6 (76.9) | 12.6 (86.3) | 12.2 (84.0) | 14.6 (91.0) | 12.5 (85.7) | 14.9 (91.7) |
| **70-74** | 10.1 (92.2) | 9.3 (84.9) | 9.6 (75.4) | 9.5 (84.8) | 9.8 (86.4) | 11.2 (91.4) | 9.7 (86.0) | 11.5 (91.0) |
| **75-79** | 6.9 (91.2) | 6.2 (82.6) | 7.4 (80.4) | 6.5 (89.7) | 6.7 (90.0) | 7.6 (93.1) | 6.5 (83.1) | 8.2 (92.0) |
| **80+** | 4.5 (81.0) | 4.4 (81.6) | 5.7 (84.4) | 4.5 (88.6) | 4.7 (90.6) | 5.5 (93.3) | 4.9 (86.7) | 6.0 (90.3) |

**Note**: Percentage of diabetes-free life expectancy in the parenthesis

**STable 7**: Either hypertension or diabetes-free life expectancy by Division in Bangladesh, 2018

| **Age** | **Barishal** | **Chattogram** | **Dhaka** | **Khulna** | **Rajshahi** | **Rangpur** | **Sylhet** | **Mymensingh** |
| --- | --- | --- | --- | --- | --- | --- | --- | --- |
| **0** | 47.7 (66.8) | 47.3 (67.7) | 50.6 (67.4) | 51.0 (69.9) | 50.3 (70.3) | 51.4 (70.5) | 48.8 (69.7) | 54.8 (74.2) |
| **1-4** | 47.8 (66.3) | 47.9 (67.2) | 50.5 (66.9) | 50.9 (69.5) | 50.8 (69.9) | 51.8 (70.1) | 49.3 (69.3) | 55.3 (73.9) |
| **5-9** | 44.0 (64.3) | 44.2 (65.3) | 46.7 (65.1) | 47.1 (67.7) | 47.1 (68.2) | 48.1 (68.3) | 45.6 (67.5) | 51.6 (72.4) |
| **10-14** | 39.2 (61.5) | 39.4 (62.5) | 41.8 (62.5) | 42.2 (65.2) | 42.1 (65.7) | 43.2 (65.9) | 40.8 (64.9) | 46.7 (70.3) |
| **15-19** | 34.3 (58.3) | 34.5 (59.3) | 36.9 (59.5) | 37.3 (62.3) | 37.2 (62.8) | 38.3 (63.1) | 35.9 (61.8) | 41.7 (67.9) |
| **20-24** | 30.1 (55.5) | 30.1 (56.4) | 32.6 (56.9) | 32.9 (59.8) | 32.5 (59.4) | 34.0 (60.7) | 31.8 (59.3) | 37.2 (65.8) |
| **25-29** | 26.2 (52.8) | 25.9 (53.3) | 28.4 (54.1) | 28.5 (56.7) | 28.5 (57.1) | 29.7 (57.9) | 27.3 (56.1) | 33.1 (63.8) |
| **30-34** | 22.1 (49.2) | 22.1 (50.4) | 24.3 (50.9) | 24.3 (53.5) | 24.2 (53.9) | 25.7 (55.2) | 23.6 (53.7) | 28.8 (61.2) |
| **35-39** | 18.4 (46.1) | 18.7 (47.6) | 20.6 (48.0) | 20.8 (51.1) | 20.4 (50.7) | 22.2 (53.1) | 19.8 (50.4) | 24.8 (58.7) |
| **40-44** | 15.5 (43.8) | 16.0 (46.2) | 17.5 (46.0) | 17.3 (48.0) | 16.9 (47.5) | 19.1 (51.4) | 16.2 (47.0) | 21.7 (57.5) |
| **45-49** | 12.5 (40.6) | 13.2 (44.2) | 14.8 (44.0) | 14.5 (46.4) | 13.7 (44.4) | 16.1 (49.7) | 13.3 (44.1) | 18.5 (55.9) |
| **50-54** | 10.1 (38.5) | 11.2 (43.2) | 12.4 (42.4) | 11.8 (44.0) | 10.9 (41.5) | 13.5 (48.4) | 10.8 (42.0) | 15.0 (52.2) |
| **55-59** | 7.9 (35.9) | 9.3 (42.9) | 10.1 (40.7) | 9.5 (42.0) | 9.1 (40.9) | 11.3 (47.3) | 8.4 (38.7) | 12.3 (50.4) |
| **60-64** | 6.1 (33.7) | 7.7 (42.7) | 7.7 (37.4) | 7.6 (40.6) | 7.0 (38.1) | 9.3 (47.2) | 6.5 (36.0) | 10.0 (48.8) |
| **65-69** | 4.6 (31.8) | 6.2 (43.6) | 6.0 (36.7) | 5.6 (38.1) | 5.1 (34.7) | 7.8 (48.8) | 5.1 (34.9) | 7.7 (47.2) |
| **70-74** | 3.5 (32.1) | 4.4 (40.5) | 4.7 (37.2) | 3.5 (31.3) | 3.7 (32.5) | 6.5 (52.8) | 3.6 (32.2) | 6.0 (47.5) |
| **75-79** | 1.7 (22.7) | 2.5 (33.6) | 3.7 (40.4) | 1.9 (25.7) | 2.9 (39.1) | 4.0 (49.2) | 2.4 (30.2) | 4.0 (44.5) |
| **80+** | 0.8 (14.3) | 1.3 (24.0) | 1.9 (27.4) | 1.5 (29.5) | 1.5 (29.0) | 2.8 (48.3) | 1.5 (26.7) | 3.0 (45.2) |

**Note**: Percentage of either hypertension or diabetes-free life expectancy in the parenthesis

**STable 8**: Both hypertension and diabetes-free life expectancy by Division in Bangladesh, 2018

| **Age** | **Barishal** | **Chattogram** | **Dhaka** | **Khulna** | **Rajshahi** | **Rangpur** | **Sylhet** | **Mymensingh** |
| --- | --- | --- | --- | --- | --- | --- | --- | --- |
| **0** | 68.5 (96.0) | 65.5 (93.8) | 70.2 (93.5) | 69.6 (95.4) | 68.9 (96.3) | 70.9 (97.2) | 66.7 (95.4) | 71.6 (97.0) |
| **1-4** | 69.1 (95.9) | 66.7 (93.7) | 70.5 (93.5) | 69.8 (95.4) | 69.9 (96.2) | 71.9 (97.2) | 67.9 (95.4) | 72.5 (96.9) |
| **5-9** | 65.4 (95.7) | 63.1 (93.3) | 66.8 (93.1) | 66.1 (95.1) | 66.3 (96.0) | 68.3 (97.0) | 64.3 (95.1) | 68.9 (96.7) |
| **10-14** | 60.7 (95.4) | 58.4 (92.8) | 61.9 (92.6) | 61.3 (94.7) | 61.4 (95.7) | 63.4 (96.8) | 59.5 (94.7) | 64.0 (96.5) |
| **15-19** | 55.9 (95.0) | 53.5 (92.1) | 57.1 (92.0) | 56.4 (94.3) | 56.5 (95.4) | 58.6 (96.5) | 54.6 (94.2) | 59.1 (96.2) |
| **20-24** | 51.3 (94.5) | 48.8 (91.4) | 52.3 (91.3) | 51.7 (93.8) | 51.9 (94.9) | 53.9 (96.2) | 50.2 (93.7) | 54.3 (95.9) |
| **25-29** | 46.6 (94.1) | 44.1 (90.6) | 47.5 (90.5) | 46.8 (93.2) | 47.1 (94.4) | 49.2 (95.8) | 45.3 (93.1) | 49.5 (95.5) |
| **30-34** | 41.9 (93.4) | 39.5 (89.9) | 42.8 (89.6) | 42.0 (92.5) | 42.2 (93.8) | 44.4 (95.4) | 40.7 (92.5) | 44.7 (95.2) |
| **35-39** | 37.3 (93.1) | 35.0 (89.0) | 38.0 (88.6) | 37.4 (92.0) | 37.5 (93.3) | 39.7 (95.1) | 36.0 (91.7) | 40.1 (94.9) |
| **40-44** | 32.7 (92.7) | 30.5 (88.1) | 33.5 (87.9) | 32.9 (91.4) | 33.0 (92.7) | 35.1 (94.6) | 31.3 (90.8) | 35.8 (94.7) |
| **45-49** | 28.1 (91.5) | 25.9 (86.6) | 29.5 (87.8) | 28.6 (91.0) | 28.2 (91.8) | 30.5 (94.3) | 27.3 (90.8) | 31.3 (94.7) |
| **50-54** | 23.9 (90.8) | 22.4 (86.7) | 25.6 (87.5) | 24.3 (90.3) | 24.1 (91.5) | 26.2 (94.0) | 23.4 (90.5) | 27.3 (94.9) |
| **55-59** | 20.2 (91.2) | 18.8 (86.6) | 21.6 (87.0) | 20.6 (91.0) | 20.4 (91.3) | 22.3 (93.3) | 19.8 (90.7) | 23.2 (94.9) |
| **60-64** | 16.8 (92.3) | 15.5 (86.8) | 17.7 (86.6) | 17.0 (91.2) | 16.7 (90.9) | 18.4 (93.5) | 16.4 (91.1) | 19.4 (94.9) |
| **65-69** | 13.5 (93.1) | 12.5 (88.0) | 14.3 (87.0) | 13.2 (89.9) | 13.2 (90.7) | 14.8 (92.7) | 13.3 (91.4) | 15.6 (95.8) |
| **70-74** | 10.4 (94.7) | 10.0 (90.8) | 11.2 (87.6) | 9.9 (87.6) | 10.3 (90.9) | 11.4 (93.1) | 10.5 (92.5) | 12.1 (95.9) |
| **75-79** | 7.3 (95.6) | 6.7 (89.0) | 8.2 (89.3) | 6.6 (90.7) | 7.1 (94.4) | 7.8 (94.7) | 7.1 (91.0) | 8.7 (96.6) |
| **80+** | 5.0 (90.5) | 4.6 (85.7) | 6.2 (91.9) | 4.7 (90.9) | 4.8 (93.8) | 5.6 (96.6) | 5.3 (93.3) | 6.2 (93.5) |

**Note**: Percentage of both hypertension and diabetes-free life expectancy in the parenthesis

**STable 9**: Age-specific mortality rate (%) by place of residence in Bangladesh, 2018

|  | **Rural** | | | **Urban** | | | **Total** | | |
| --- | --- | --- | --- | --- | --- | --- | --- | --- | --- |
| **Age** | **Male** | **Female** | **Total** | **Male** | **Female** | **Total** | **Male** | **Female** | **Total** |
| **0-1** | 2.97 | 2.65 | 2.82 | 2.62 | 2.51 | 2.57 | 2.83 | 2.59 | 2.71 |
| **1-5** | 0.30 | 0.21 | 0.26 | 0.13 | 0.11 | 1.22 | 0.23 | 0.17 | 0.20 |
| **5-10** | 0.08 | 0.05 | 0.07 | 0.08 | 0.04 | 0.06 | 0.08 | 0.05 | 0.06 |
| **10-15** | 0.05 | 0.07 | 0.06 | 0.04 | 0.03 | 0.03 | 0.05 | 0.05 | 0.05 |
| **15-19** | 0.13 | 0.13 | 0.13 | 0.12 | 0.09 | 0.11 | 0.13 | 0.11 | 0.12 |
| **20-24** | 0.11 | 0.11 | 0.11 | 0.06 | 0.05 | 0.06 | 0.09 | 0.08 | 0.09 |
| **25-29** | 0.13 | 0.11 | 0.12 | 0.08 | 0.06 | 0.07 | 0.11 | 0.08 | 0.09 |
| **30-34** | 0.12 | 0.15 | 0.14 | 0.09 | 0.09 | 0.09 | 0.10 | 0.12 | 0.11 |
| **35-39** | 0.18 | 0.14 | 0.16 | 0.14 | 0.13 | 0.13 | 0.16 | 0.13 | 0.15 |
| **40-44** | 0.20 | 0.21 | 0.20 | 0.27 | 0.18 | 0.23 | 0.24 | 0.20 | 0.22 |
| **45-49** | 0.59 | 0.38 | 0.49 | 0.38 | 0.42 | 0.40 | 0.49 | 0.40 | 0.45 |
| **50-54** | 0.85 | 0.55 | 0.69 | 0.88 | 0.48 | 0.67 | 0.86 | 0.52 | 0.68 |
| **55-59** | 1.09 | 0.64 | 0.86 | 1.28 | 0.76 | 1.04 | 1.18 | 0.69 | 0.94 |
| **60-64** | 1.56 | 1.24 | 1.41 | 1.57 | 1.22 | 1.41 | 1.57 | 1.23 | 1.41 |
| **65-69** | 2.59 | 1.79 | 2.20 | 3.04 | 1.93 | 2.53 | 2.78 | 1.84 | 2.33 |
| **70-74** | 2.56 | 2.67 | 2.61 | 3.45 | 2.43 | 2.96 | 2.91 | 2.58 | 2.75 |
| **75-79** | 9.34 | 7.35 | 8.39 | 7.41 | 8.28 | 7.84 | 8.64 | 7.70 | 8.19 |
| **80+** | 11.99 | 9.81 | 10.78 | 12.29 | 10.39 | 11.21 | 12.09 | 10.02 | 10.93 |

**STable 10**: Age-specific hypertension and diabetes rate (%) by place of residence in Bangladesh, 2018

| **Age** | **Hypertension** | | **Diabetes** | |
| --- | --- | --- | --- | --- |
|  | **Rural** | **Urban** | **Rural** | **Urban** |
|  | **Total (Male, Female)** | **Total (Male, Female)** | **Total (Male, Female)** | **Total (Male, Female)** |
| **18-19** | 7.2 (11.737, 4.9) | 5.8 (6.9, 4.7) | 4.5 (6.8, 3.3) | 2.1 (2.7, 1.9) |
| **20-24** | 9.7 (11.881, 8.5) | 9.0 (15.0, 5.9) | 2.7 (2.6, 2.6) | 7.9 (10.6, 6.5) |
| **25-29** | 12.7 (10.664, 13.9) | 13.9 (13.3, 14.3) | 3.9 (3.9, 3.7) | 7.5 (2.9, 10.6) |
| **30-34** | 18.5 (15.461, 20.2) | 21.8 (19.0, 24.0) | 6.9 (5.5, 7.7) | 9.6 (9.8, 9.0) |
| **35-39** | 25.7 (20.807, 29.8) | 30.1 (25.0, 34.8) | 8.0 (6.2, 9.5) | 15.7 (14.4, 17.1) |
| **40-44** | 28.8 (19.945, 35.8) | 34.4 (27.2, 41.1) | 10.6 (12.9, 9.0) | 19.9 (16.1, 23.6) |
| **45-49** | 34.7 (25.731, 41.5) | 42.7 (35.4, 48.8) | 12.0 (10.2, 13.4) | 18.6 (15.8, 20.1) |
| **50-54** | 39.6 (31.834, 48.3) | 44.4 (43.7, 45.2) | 16.3 (17.7, 15.2) | 21.2 (19.4, 24.7) |
| **55-59** | 42.5 (34.800, 48.9) | 52.9 (46.3, 58.1) | 12.6 (12.1, 13.0) | 25.5 (19.7, 30.5) |
| **60-64** | 47.2 (41.404, 53.0) | 53.4 (50.5, 57.6) | 16.2 (18.5, 13.9) | 19.7 (18.8, 21.0) |
| **65-69** | 51.5 (50.279, 52.7) | 50.0 (40.7, 65.6) | 14.2 (16.9, 11.2) | 17.9 (16.7, 22.6) |
| **70-74** | 49.3 (49.754, 48.5) | 62.3 (56.5, 71.9) | 15.7 (15.9, 15.6) | 25.3 (34.1, 12.9) |
| **74-79** | 49.6 (41.667, 61.2) | 65.6 (60.0, 70.6) | 11.1 (9.9, 12.8) | 27.6 (28.6, 21.4) |
| **80+** | 61.9 (57.265, 66.7) | 69.7 (61.5, 75.0) | 10.5 (13.8, 7.2) | 22.7 (21.4, 23.7) |

[Note: Prevalence data was only available from age group 18 in BDHS. ^48^ Prevalence of ages 0-17 had not been reported because of lower prevalence reported by past studies.^49,50^]

**STable 11**: Hypertension, diabetes, either and both free life expectancy by place of residence in Bangladesh, 2018

| **Age** | **Overall, T (M, F)** | | **Hypertension-free, T (M, F)** | | **Diabetes-free, T (M, F)** | | **Either-free, T (M, F)** | | **Both-free, T (M, F)** | |
| --- | --- | --- | --- | --- | --- | --- | --- | --- | --- | --- |
|  | **Rural** | **Urban** | **Rural** | **Urban** | **Rural** | **Urban** | **Rural** | **Urban** | **Rural** | **Urban** |
| **0** | 72.0 (70.8, 73.3) | 71.8 (71.3, 73.9) | 53.9 (55.5, 52.4) | 51.0 (53.0, 49.7) | 66.2 (64.9, 67.7) | 62.5 (62.7, 63.8) | 50.9 (51.9, 49.9) | 46.6 (48.5, 45.3) | 69.1 (68.2, 70.1) | 66.7 (67.0, 69.7) |
| **1-4** | 72.9 (74.2, 71.8) | 72.6 (72.1, 74.7) | 54.4 (56.1, 52.8) | 51.3 (53.3, 49.9) | 67.0 (65.8, 68.5) | 63.0 (63.3, 64.4) | 51.3 (52.4, 50.2) | 46.7 (48.8, 45.4) | 70.0 (69.2, 70.9) | 67.4 (67.7, 70.5) |
| **5-9** | 69.1 (70.4, 68.0) | 69.4 (68.2, 70.8) | 50.5 (52.2, 48.9) | 47.9 (49.4, 46.0) | 63.2 (62.0, 64.6) | 59.8 (59.4, 60.4) | 47.4 (48.6, 46.3) | 43.3 (44.8, 41.4) | 66.2 (65.4, 67.1) | 64.2 (63.8, 66.5) |
| **10-14** | 64.4 (65.6, 63.3) | 64.6 (63.5, 65.9) | 45.7 (47.4, 44.0) | 43.0 (44.6, 41.1) | 58.4 (57.2, 59.8) | 54.9 (54.7, 55.6) | 42.6 (43.8, 41.4) | 38.4 (40.0, 36.5) | 61.4 (60.7, 62.3) | 59.3 (59.1, 61.7) |
| **15-19** | 59.5 (60.8, 58.4) | 59.7 (58.6, 61.0) | 40.8 (42.5, 39.2) | 38.1 (39.6, 36.1) | 53.6 (52.4, 55.0) | 50.0 (49.8, 50.6) | 37.7 (38.9, 36.5) | 33.4 (35.1, 31.6) | 56.6 (55.8, 57.5) | 54.4 (54.2, 56.7) |
| **20-24** | 54.9 (56.2, 53.8) | 55.0 (53.9, 56.3) | 36.4 (38.4, 34.6) | 33.6 (35.2, 31.5) | 49.2 (48.0, 50.5) | 45.4 (45.2, 46.0) | 33.5 (35.0, 32.1) | 29.0 (30.7, 27.1) | 51.9 (51.2, 52.8) | 49.7 (49.5, 52.0) |
| **25-29** | 50.2 (51.5, 49.1) | 50.2 (49.1, 51.4) | 32.1 (34.2, 30.3) | 29.1 (31.1, 26.9) | 44.5 (43.4, 45.9) | 40.9 (40.8, 41.4) | 29.3 (30.9, 27.9) | 24.8 (26.9, 22.7) | 47.2 (46.5, 48.1) | 44.9 (44.9, 47.1) |
| **30-34** | 45.5 (46.7, 44.4) | 45.3 (44.3, 46.6) | 27.9 (29.9, 26.1) | 24.9 (26.8, 22.7) | 40.0 (38.9, 41.3) | 36.4 (36.1, 37.0) | 25.2 (26.7, 23.8) | 20.9 (22.8, 18.9) | 42.5 (41.8, 43.4) | 40.2 (40.1, 42.3) |
| **35-39** | 40.8 (42.1, 39.6) | 40.5 (39.4, 41.8) | 24.0 (25.9, 22.3) | 21.1 (22.9, 19.0) | 35.6 (34.4, 37.0) | 32.1 (31.8, 32.6) | 21.5 (22.9, 20.2) | 17.4 (19.2, 15.5) | 37.9 (37.1, 38.8) | 35.5 (35.3, 37.5) |
| **40-44** | 36.1 (37.3, 35.0) | 35.8 (34.7, 37.0) | 20.5 (22.1, 18.9) | 17.7 (19.3, 15.8) | 31.3 (30.0, 32.7) | 28.0 (27.7, 28.7) | 18.2 (19.3, 17.1) | 14.6 (16.1, 12.9) | 33.3 (32.5, 34.3) | 31.0 (30.7, 32.8) |
| **45-49** | 31.4 (32.7, 30.3) | 31.2 (30.1, 32.3) | 17.1 (18.3, 15.9) | 14.6 (15.9, 13.0) | 27.1 (25.9, 28.4) | 24.3 (23.8, 25.1) | 15.2 (16.0, 14.3) | 11.9 (13.1, 10.6) | 28.8 (28.0, 29.8) | 26.8 (26.5, 28.7) |
| **50-54** | 27.2 (28.3, 26.1) | 26.7 (25.7, 28.0) | 14.2 (15.1, 13.3) | 12.0 (12.9, 10.7) | 23.3 (22.1, 24.6) | 20.7 (20.1, 21.6) | 12.5 (13.0, 11.9) | 9.7 (10.4, 8.8) | 24.8 (24.0, 25.7) | 22.9 (22.5, 24.8) |
| **55-59** | 23.0 (24.0, 22.1) | 22.6 (21.7, 23.6) | 11.7 (12.3, 11.0) | 9.6 (10.6, 8.2) | 19.9 (18.9, 21.0) | 17.4 (16.8, 18.3) | 10.3 (10.7, 9.9) | 7.7 (8.4, 6.9) | 21.0 (20.3, 21.9) | 19.2 (18.9, 21.2) |
| **60-64** | 18.9 (19.7, 18.2) | 18.6 (18.0, 19.4) | 9.2 (9.6, 8.8) | 7.7 (8.6, 6.4) | 16.3 (15.4, 17.3) | 14.5 (13.8, 15.5) | 8.1 (8.3, 7.7) | 6.2 (6.7, 5.6) | 17.3 (16.6, 18.1) | 15.9 (15.5, 17.5) |
| **65-69** | 15.1 (15.8, 14.5) | 14.8 (14.3, 15.5) | 7.2 (7.3, 6.9) | 5.8 (6.7, 4.6) | 13.1 (12.4, 13.9) | 11.4 (10.7, 12.4) | 6.3 (6.4, 6.1) | 4.8 (5.1, 4.3) | 13.9 (13.2, 14.6) | 12.4 (12.2, 14.1) |
| **70-74** | 11.6 (12.0, 11.2) | 11.5 (11.2, 11.8) | 5.5 (5.7, 5.1) | 4.0 (4.6, 3.3) | 10.1 (9.7, 10.6) | 8.6 (8.0, 9.6) | 4.8 (5.0, 4.5) | 3.1 (3.3, 2.9) | 10.7 (10.3, 11.1) | 9.5 (9.2, 10.7) |
| **75-79** | 7.9 (8.4, 7.4) | 7.9 (7.8, 8.0) | 3.5 (3.8, 3.0) | 2.6 (3.1, 2.2) | 7.0 (6.5, 7.6) | 5.9 (5.9, 6.2) | 3.0 (3.3, 2.7) | 2.2 (2.6, 1.9) | 7.4 (7.0, 7.9) | 6.4 (6.4, 7.5) |
| **80+** | 5.7 (6.1, 5.3) | 5.6 (5.3, 5.8) | 2.2 (2.3, 2.0) | 1.7 (2.0, 1.5) | 5.1 (4.6, 5.6) | 4.3 (4.1, 4.5) | 1.8 (1.8, 1.8) | 1.5 (2.0, 1.1) | 5.4 (4.9, 5.8) | 4.6 (4.4, 5.1) |

**Note:** T=total, M=Male, F= Female life expectancy
